# Modified reverse degree descriptors for combined topological and entropy characterizations of 2D metal organic frameworks: applications in graph energy prediction

**DOI:** 10.3389/fchem.2024.1470231

**Published:** 2024-09-25

**Authors:** A. R. Abul Kalaam, A. Berin Greeni, Micheal Arockiaraj

**Affiliations:** ^1^ School of Advanced Sciences, Vellore Institute of Technology, Chennai, India; ^2^ Department of Mathematics, Loyola College, Chennai, India

**Keywords:** metal organic frameworks, reverse degree based descriptors, bond-wise entropy analysis, graph energies, structure property regression models

## Abstract

Topological descriptors are widely utilized as graph theoretical measures for evaluating the physicochemical properties of organic frameworks by examining their molecular structures. Our current research validates the usage of topological descriptors in studying frameworks such as metal-butylated hydroxytoluene, NH-substituted coronene transition metal, transition metal-phthalocyanine, and conductive metal-octa amino phthalocyanine. These metal organic frameworks are crucial in nanoscale research for their porosity, adaptability, and conductivity, making them essential for advanced materials and modern technology. In this study, we provide the topological and entropy characterizations of these frameworks by employing robust reverse degree based descriptors, which offer insightful information on structural complexities. This structural information is applied to predict the graph energy of the considered metal organic frameworks using statistical regression models.

## 1 Introduction

Two-dimensional metal organic frameworks (MOFs) are revolutionizing nanoscale research with their unique blend of inorganic and organic components, offering exceptional advantages like porosity and tunability. These porous crystals feature cage-like architectures formed by aromatic organic moieties and square-planar metal ions, finding diverse applications in gas catalysis, drug delivery, sensors, optoelectronics, storage, and adsorption (Kinoshita et al., 1959; Lee et al., 2009; Horcajada et al., 2008; Murray et al., 2009). Metal-organic surfaces based on metal-butylated hydroxytoluene (MBHT) exhibit promising electronic and magnetic properties, particularly for transition metals like M = {Co, Fe, Mn, Cr} (Clough et al., 2015; Chakravarty et al., 2016a). Among MBHT-derived materials, CoBHT, FeBHT, and MnBHT display planar ferromagnetic half-metallism, while CrBHT possesses a spin-frustrated kagome lattice leading to antiferromagnetic semimetallic behavior. These frameworks exhibit high sensitivity towards gas molecules like carbon monoxide, altering their electronic and magnetic properties significantly upon adsorption. A coronene molecule substituted with an − NH group, complexed with transition metals (NHC-TM), presents a promising pathway to developing novel materials for spintronic devices (Chakravarty et al., 2016b). These MOFs which feature coronene molecules bonded to transition metals in a square planar geometry, exhibit favorable formation energy, making practical synthesis feasible and enhancing control over their magnetic and electronic properties (Dong et al., 2018).

Transition metal-phthalocyanine (TM-Pc) based MOFs exhibit captivating two-dimensional structures with distinctive electronic and magnetic features. TM-Pc is derived from the transition metal-tetracyanobenzene (TM-TCNB) framework through benzene ring rotation and on-surface polymerization, involving transition metals TM = {Ti, V, Cr, Co, Ni, Cu, Zn} (Mabrouk and Hayn, 2015). TM-Pc demonstrates superior stability over TM-TCNB by approximately 7eV per cell, revealing local energy minima in free-standing layers. Both materials, characterized by TM^2+^ states, showcase potential for spintronics (Mabrouk et al., 2018). Metal-octa amino phthalocyanine (MOAPc) shows significant promise in applications like energy storage, catalysis, and sensing due to their unique bimetallic properties. Co^2+^, Ni^2+^, and Cu^2+^ serve as both metal centers and nodes within the MOAPc lattice (Li et al., 2017). This exploration provides insights into the effects of metal substitutions, enriching the understanding of MOAPc-based MOFs for diverse applications (Park et al., 2023).

In mathematical chemistry, graph theoretical techniques are employed to study molecular structures, properties, and reactions. Topological descriptors play a crucial role in this field, serving as essential tools for analyzing complex molecular systems. These descriptors are numerical values derived from molecular structure connectivity, representing the positions of atoms and bonds within the molecule. The widespread use of distance-based indices like the Wiener index and degree-based indices such as the Zagreb indices has significantly advanced the field of topological indices (Wiener, 1947; Gutman and Trinajstić, 1972; Raza et al., 2023a; Arockiaraj et al., 2023a). Applications of degree and distance-based topological indices are creating new possibilities in drug discovery and neural network research (Zhang et al., 2022; Zhang et al., 2024; Arockiaraj et al., 2024a). In particular, the robust refinement of reverse degree indices significantly improved the correlation with the physicochemical properties of molecules and was applied to drug compounds related to coronavirus, blood cancer and cardiovascular drug compounds (Arockiaraj et al., 2023b; Arockiaraj et al., 2023c; Arockiaraj et al., 2024b). This approach develops various graph degree sequences with variable parameters, enhancing statistical models for the considered datasets. In this work, we implement the modified reverse degree method to the recently introduced hybrid topological indices (Arockiaraj et al., 2023a).

Structural entropy, introduced by Shannon, deals with unpredictability or uncertainty in datasets (Shannon, 1948; Arockiaraj et al., 2023d). It indicates micro-state diversity, reflecting various positions in a system with atoms and molecules. Higher entropy within a system implies greater disorder, signifying more potential micro-states. This principle extends to chemical structures, providing a valuable tool for analyzing their stability and structural data (Dehmer, 2008). Graph entropies link probability distributions to graph elements, such as vertices and edges, aiding in comprehensive graph analysis. These explorations aid in predicting the graph energies of MOFs using graph theoretical and statistical techniques.

Recent studies on degree-based descriptors have been instrumental in analyzing various metal organic and covalent organic frameworks, such as phthalocyanine frameworks, trans-Pd–(NH_2_)S lattice, metal butylated hydroxytoluene frameworks, and FeTPyP-CO MOFs (Nadeem et al., 2021; Azeem et al., 2021; Zaman et al., 2023; Yu et al., 2023; Al-Dayel et al., 2024). Additionally, entropy-based investigations have focused on metal phthalocyanine COFs, isoreticular metal-organic frameworks, and coronene-based MOFs (Arockiaraj et al., 2023e; Abraham et al., 2022; Manzoor et al., 2021; Raza et al., 2023b; Imran et al., 2023; Waheed et al., 2023; Ghani et al., 2022; Abul Kalaam and Berin Greeni, 2024; Yang et al., 2023). This paper presents the implementation of modified reverse degree-based descriptors for four types of MOFs, the computation of entropy measures through bond-wise comparative analysis, and the development of predictive models for graph energy.

## 2 Methodology

In this study, MOFs are displayed through two-dimensional graph structures. We denote such a two-dimensional structure by G with |V(G)| and |E(G)| indicating the number of vertices and edges in the graph G respectively. In this context, the term vertex degree, denoted as $d_{v}$ , represents the total number of vertices adjacent to vertex v . The maximum degree in graph G is denoted as Δ(G) . The reverse version of the degree (Ediz, 2015) and its generalized form (Arockiaraj et al., 2023b) have received significant attention in recent years. It is denoted as $\left(M_{k}R\left(d_{v}\right)\right)$ , incorporating a variable parameter k (k≥1) and defined as

$$M_{k}R\left(d_{v}\right)=\left\{\begin{array}{cc} \Delta \left(G\right)-d_{v}+k\; & :k\leq d_{v}\\ \Delta \left(G\right)-d_{v}+k\left(mod\;\Delta \left(G\right)\right)\; & :k>d_{v} \end{array}\right.$$

Therefore, the general form of topological descriptors (TD) for the modified reverse degree classification of metal organic frameworks is defined as follows:

$$M_{k}RTD\left(G\right)=\underset{uv\in E\left(G\right)}{\sum }M_{k}RTD\left(d_{u},d_{v}\right)=\underset{uv\in E\left(G\right)}{\sum }TD\left(M_{k}R\left(d_{u}\right),M_{k}R\left(d_{v}\right)\right)$$

Here, $TD\left(M_{k}R\left(d_{u}\right),M_{k}R\left(d_{v}\right)\right)$ denotes the topological descriptors function, ensuring symmetrical mutuality as given below.

$$TD\left(M_{k}R\left(d_{u}\right),M_{k}R\left(d_{v}\right)\right)=TD\left(M_{k}R\left(d_{v}\right),M_{k}R\left(d_{u}\right)\right)$$

Suppose the edge set of G is partitioned into equivalence classes $E\left(G\right)=\left\{E_{1}\cup E_{2}\cup \cdots \cup E_{n}\right\}$ , such that each edge in the class $E_{i}$ has the same modified degree parameters at the end vertices. Then, the modified reverse degree topological descriptor for the class $E_{i}$ is expressed in the following form with $uv\in E_{i}$ .

$$M_{k}RTD\left(E_{i}\right)=|E_{i}|\cdot TD\left(M_{k}R\left(d_{u}\right),M_{k}R\left(d_{v}\right)\right)$$

Therefore, the overall modified reverse degree descriptors for graph G is calculated by summing the individual contributions from each class $E_{i}$ .

$$M_{k}RTD\left(G\right)=\overset{n}{\underset{i=1}{\sum }}|E_{i}|\cdot TD\left(M_{k}R\left(d_{u}\right),M_{k}R\left(d_{v}\right)\right)$$

The modified reverse degree based topological descriptor functions considered in this study are stated below.

• Modified reverse first Zagreb descriptor:

$$M_{k}RM_{1}\left(d_{u},d_{v}\right)=M_{k}R\;\left(d_{u}\right)+M_{k}R\;\left(d_{v}\right)$$ (1)

    • Modified reverse second Zagreb descriptor:

$$M_{k}RM_{2}\left(d_{u},d_{v}\right)=M_{k}R\;\left(d_{u}\right)\times M_{k}R\;\left(d_{v}\right)$$ (2)

    • Modified reverse forgotten descriptor:

$$M_{k}RF\left(d_{u},d_{v}\right)={\left(M_{k}R\;\left(d_{u}\right)\right)}^{2}+{\left(M_{k}R\;\left(d_{v}\right)\right)}^{2}$$ (3)

    • Modified reverse hyper-Zagreb descriptor:

$$M_{k}RHZ\left(d_{u},d_{v}\right)={\left(M_{k}R\;\left(d_{u}\right)+M_{k}R\;\left(d_{v}\right)\right)}^{2}$$ (4)

    • Modified reverse third redefined Zagreb descriptor:

$$M_{k}RReZ_{3}\left(d_{u},d_{v}\right)=\left(M_{k}R\;\left(d_{u}\right)+M_{k}R\;\left(d_{v}\right)\right)\times \left(M_{k}R\;\left(d_{u}\right)\times M_{k}R\;\left(d_{v}\right)\right)$$ (5)

    • Modified reverse bi-Zagreb descriptor:

$$M_{k}RBM\left(d_{u},d_{v}\right)=\left(M_{k}R\;\left(d_{u}\right)+M_{k}R\;\left(d_{v}\right)+\left(M_{k}R\;\left(d_{u}\right)\times M_{k}R\;\left(d_{v}\right)\right)\right)$$ (6)

    • Modified reverse tri-Zagreb descriptor:

$$M_{k}RBM\left(d_{u},d_{v}\right)=\left({\left(M_{k}R\;\left(d_{u}\right)\right)}^{2}+{\left(M_{k}R\;\left(d_{v}\right)\right)}^{2}+\left(M_{k}R\;\left(d_{u}\right)\times M_{k}R\;\left(d_{v}\right)\right)\right)$$ (7)

    • Modified reverse bi-Zagreb harmonic descriptor:

$$M_{k}RBMH\left(d_{u},d_{v}\right)=\frac{\left(M_{k}R\;\left(d_{u}\right)+M_{k}R\;\left(d_{v}\right)+\left(M_{k}R\;\left(d_{u}\right)\times M_{k}R\;\left(d_{v}\right)\right)\right)\times \left(M_{k}R\;\left(d_{u}\right)+M_{k}R\;\left(d_{v}\right)\right)}{2}$$ (8)

• Modified reverse tri-Zagreb harmonic descriptor:

$$M_{k}RTMH\left(d_{u},d_{v}\right)=\frac{\left({\left(M_{k}R\;\left(d_{u}\right)\right)}^{2}+{\left(M_{k}R\;\left(d_{v}\right)\right)}^{2}+\left(M_{k}R\;\left(d_{u}\right)\times M_{k}R\;\left(d_{v}\right)\right)\right)\times M_{k}R\;\left(d_{u}\right)+M_{k}R\;\left(d_{v}\right)}{2}$$ (9)

## 3 Computation of modified reverse degree metrics

**FIGURE 1:**
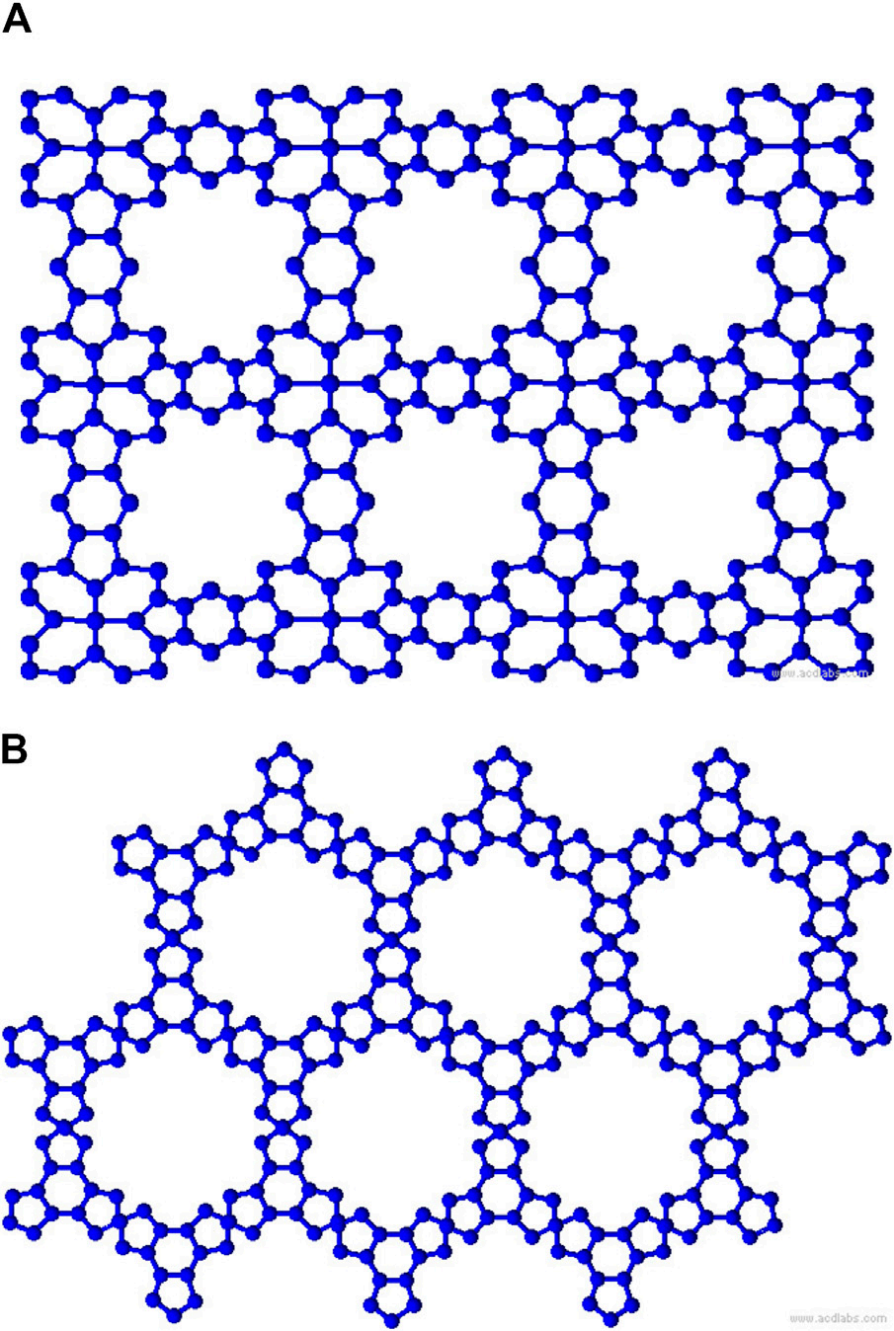
MOFs of dimensions (2,3) **(A)** TM-Pc and **(B)** MBHT.

**FIGURE 2:**
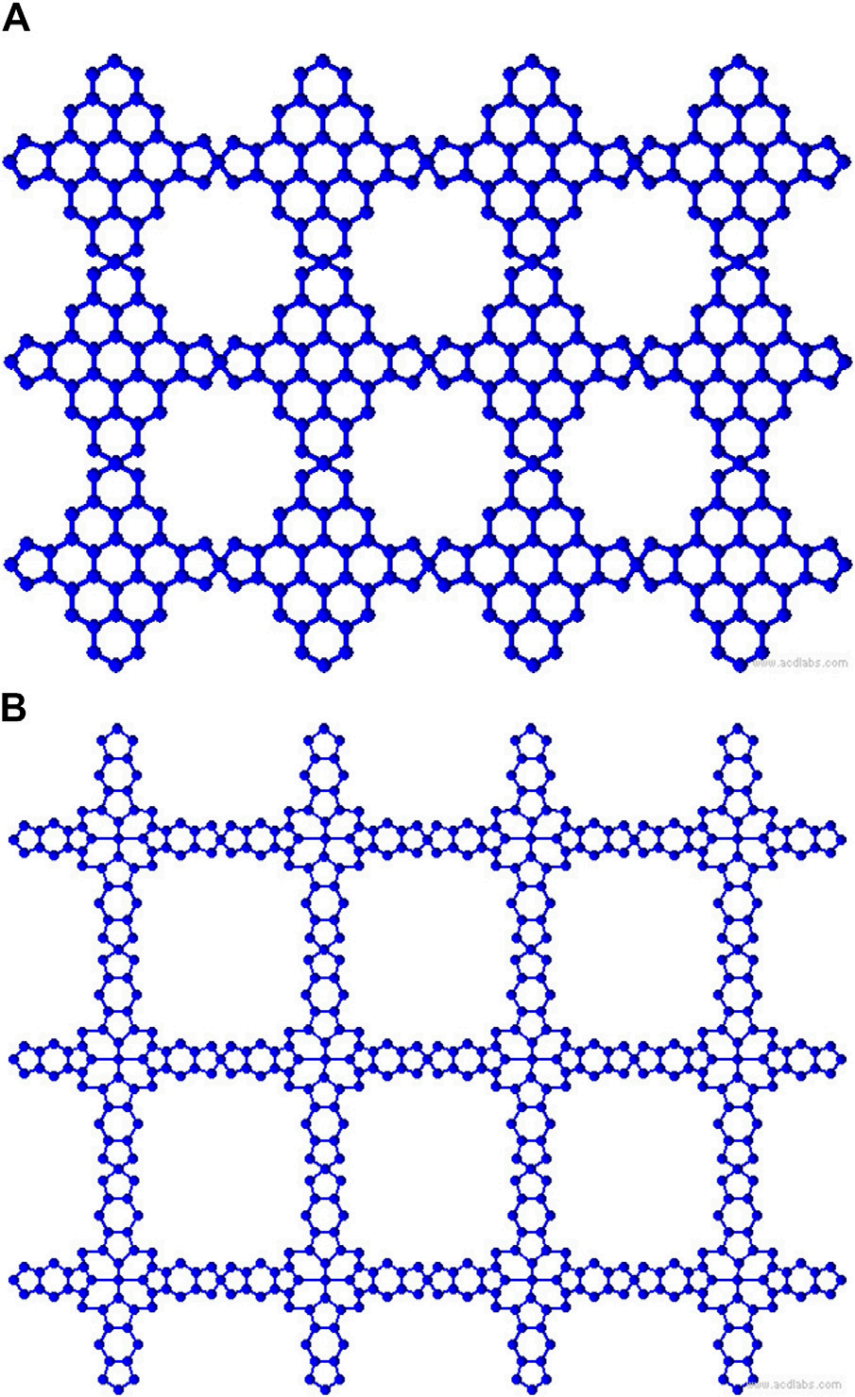
MOFs of dimensions (2,3) **(A)** NHC-TM and **(B)** MOAPc.

Metal organic frameworks based on transition metal-phthalocyanine, conductive metal-octa amino phthalocyanine, metal-butylated hydroxytoluene, and NH-substituted coronene transition metal are respectively denoted as TM-Pc (m,n) , MOAPc (m,n) , MBHT (m,n) and NHC-TM (m,n) where m and n representing the void space in the rows and columns, as dimensions (2,3) shown in Figures 1, 2. The graph representations have the following properties: for TM-Pc (m,n) , |V(G)|=29mn+23(m+n)+17 and |E(G)|=40mn+30(m+n)+20 ; for MOAPc (m,n) , |V(G)|=51mn+52(m+n)+53 and |E(G)|=68mn+68m+68n+68 ; for MBHT (m,n) , |V(G)|=27mn+28(m+n)+1 and |E(G)|=36mn+36m+36n ; and for NHC-TM (m,n) , |V(G)|=34mn+35(m+n)+36 and |E(G)|=46mn+46m+46n+46 . From these MOFs, we observe that they have a maximum degree of 4. Hence, the modified reverse degrees of the vertices are presented below.

$$M_{1}R\left(d_{v}\right)=\left\{\begin{array}{cc} 4\; & :\;d_{v}=1\\ 3\; & :\;d_{v}=2\\ 2\; & :\;d_{v}=3\\ 1\; & :\;d_{v}=4 \end{array}\right.$$

$$M_{2}R\left(d_{v}\right)=\left\{\begin{array}{cc} 1\; & :\;d_{v}=1\\ 4\; & :\;d_{v}=2\\ 3\; & :\;d_{v}=3\\ 2\; & :\;d_{v}=4 \end{array}\right.$$

$$M_{3}R\left(d_{v}\right)=\left\{\begin{array}{cc} 2\; & :\;d_{v}=1\\ 1\; & :\;d_{v}=2\\ 4\; & :\;d_{v}=3\\ 3\; & :\;d_{v}=4 \end{array}\right.$$

To compute the bond-additive modified reverse degree-based topological descriptors, we need the edge partitions of MOFs based on the normal degrees, which are presented in Table 1. Given the complexity of computing each descriptor and variable parameter, we illustrate the first Zagreb descriptor in Equation 1 with the modified reverse variable k=1 for the TM-Pc framework. The normal degree classes (2,2), (2,3), (3,3), and (3,4) are modified into (3,3), (3,2), (2,2), and (2,1), respectively, when the reverse variable is fixed to k=1 . Therefore,

$$\begin{array}{l} M_{1}RM_{1}\left(\text{TM}-\text{Pc}\left(m,n\right)\right)=\left(4\left(m+n+2\right)\right)\cdot \left(3+3\right)\\ \;+\left(16mn+12+\left(m+n\right)+8\right)\\ \;\cdot \left(3+2\right)+\left(20mn+10\left(m+n\right)\right)\\ \;\cdot \left(2+2\right)+\left(4\left(mn+m+n+1\right)\right)\\ \;\cdot \left(2+1\right)\\ M_{1}RM_{1}\left(\text{TM}-\text{Pc}\left(m,n\right)\right)=172mn+136\left(m+n\right)+100 \end{array}$$

For k=2 , the normal degree classes (2,2), (2,3), (3,3), and (3,4) are modified to (4,4), (4,3), (3,3), and (3,2) respectively. Therefore,

$$\begin{array}{l} M_{2}RM_{1}\left(\text{TM}-\text{Pc}\left(m,n\right)\right)=\left(4\left(m+n+2\right)\right)\cdot \left(4+4\right)\\ \;+\left(16mn+12+\left(m+n\right)+8\right)\\ \;\cdot \left(4+3\right)\;+\left(20mn+10\left(m+n\right)\right)\\ \;\cdot \left(3+3\right)+\left(4\left(mn+m+n+1\right)\right)\\ \;\cdot \left(3+2\right)\\ M_{2}RM_{1}\left(\text{TM}-\text{Pc}\left(m,n\right)\right)=252mn+196\left(m+n\right)+140 \end{array}$$

Similarly for k=3 , the normal degree classes (2,2), (2,3), (3,3), and (3,4) are modified to (1,1), (1,4), (4,4), and (4,3) respectively. Hence,

$$\begin{array}{l} M_{3}RM_{1}\left(\text{TM}-\text{Pc}\left(m,n\right)\right)=\left(4\left(m+n+2\right)\right)\cdot \left(1+1\right)\\ \;+\left(16mn+12+\left(m+n\right)+8\right)\\ \;\cdot \left(1+4\right)\;+\left(20mn+10\left(m+n\right)\right)\\ \;\cdot \left(4+4\right)+\left(4\left(mn+m+n+1\right)\right)\\ \;\cdot \left(4+3\right)\\ M_{3}RM_{1}\left(\text{TM}-\text{Pc}\left(m,n\right)\right)=268mn+176\left(m+n\right)+84 \end{array}$$

Using the modified reverse topological descriptors detailed in Equations 1–9 and the edge partitions in Table 1, we computed the topological indices for MOFs, which are systematically presented in Tables 2–5 for reversing parameters k=1,2, and 3 .

**TABLE 1:** Edge partitions of MOFs based on vertex degrees.

MOFs	(2,2)	(2,3)	(2,4)	(3,3)	(3,4)
TM-Pc	4(m+n+2)	16mn+12(m+n)+8	—	20mn+10(m+n)	4(mn+m+n+1)
MOAPc	4(m+n+2)	32(mn+m+n+1)	4(2mn+m+n)	24(mn+m+n+1)	4(mn+m+n+1)
MBHT	4(m+n+1)	12(mn+m+n)	12mn+8(m+n)−4	12(mn+m+n)	—
NHC-TM	4(m+n+2)	16(mn+m+n+1)	4(2mn+m+n)	22(mn+m+n+1)	—

**TABLE 2:** Modified degree descriptors for TM-Pc MOF with reversing parameters k =1, 2, 3.

TM-Pc based MOFs			
TD	k=1	k=2	k=3
$M_{k}RM_{1}$	172*mn* + 136(*m* + *n*) + 100	252*mn* + 196(*m* + *n*) + 140	268*mn* + 176(*m* + *n*) + 84
$M_{k}RM_{2}$	$184mn+156\left(m+n\right)+128$	$396mn+322\left(m+n\right)+248$	$432mn+260\left(m+n\right)+88$
$M_{k}RF$	$388mn+328\left(m+n\right)+268$	$812mn+660\left(m+n\right)+508$	$1012mn+632\left(m+n\right)+252$
$M_{k}RHZ$	$756mn+640\left(m+n\right)+524$	$1604mn+1304\left(m+n\right)+1004$	$1876mn+1152\left(m+n\right)+428$
$M_{k}RReZ_{3}$	$824mn+760\left(m+n\right)+696$	$2544mn+2180\left(m+n\right)+1816$	$3216mn+1864\left(m+n\right)+512$
$M_{k}RBM$	$356mn+292\left(m+n\right)+228$	$648mn+518\left(m+n\right)+388$	$700mn+436\left(m+n\right)+172$
$M_{k}RTM$	$572mn+484\left(m+n\right)+396$	$1208mn+982\left(m+n\right)+756$	$1444mn+892\left(m+n\right)+340$
$M_{k}RBMH$	$790mn+700\left(m+n\right)+610$	$2074mn+1742\left(m+n\right)+1410$	$2546mn+1508\left(m+n\right)+470$
$M_{k}RTMH$	$1282mn+1176\left(m+n\right)+1070$	$3882mn+3322\left(m+n\right)+2762$	$5198mn+3080\left(m+n\right)+962$

**TABLE 3:** Modified reverse degree descriptors for MOAPc MOFs with reversing parameters k =1, 2, 3.

MOAPc based MOFs			
TD	k=1	k=2	k=3
$M_{k}RM_{1}$	320m+320n+324mn+316	444m+444n+436mn+452	404m+404n+412mn+396
$M_{k}RM_{2}$	380m+380n+392mn+368	720m+720n+688mn+752	576m+576n+584mn+568
$M_{k}RF$	800m+800n+828mn+772	1492m+1492n+1444mn+1540	1460m+1460n+1492mn+1428
$M_{k}RHZ$	1560m+1560n+1612mn+1508	2932m+2932n+2820mn+3044	2612m+2612n+2660mn+2564
$M_{k}RReZ_{3}$	1920m+1920n+2040mn+1800	4808m+4808n+4488mn+5128	4104m+4104n+4144mn+4064
$M_{k}RBM$	700m+700n+716mn+684	1164m+1164n+1124mn+1204	980m+980n+996mn+964
$M_{k}RTM$	1180m+1180n+1220mn+1140	2212m+2212n+2132mn+2292	2036m+2036n+2076mn+1996
$M_{k}RBMH$	1740m+1740n+1826mn+1654	3870m+3870n+3654mn+4086	3358m+3358n+3402mn+3314
$M_{k}RTMH$	2980m+2980n+3174mn+2786	7382m+7382n+6950mn+7814	6922m+6922n+7014mn+6830

**TABLE 4:** Modified reverse degree descriptors for MBHT MOFs with reversing parameters k =1, 2, 3.

MBHT based MOFs			
TD	k=1	k=2	k=3
$M_{k}RM_{1}$	188m+188n+192mn−4	236m+236n+228mn+8	196m+196n+204mn−8
$M_{k}RM_{2}$	252m+252n+264mn−12	380m+380n+348mn+32	268m+268n+276mn−8
$M_{k}RF$	524m+524n+552mn−28	804m+804n+756mn+48	676m+676n+708mn−32
$M_{k}RHZ$	1028m+1028n+1080mn−52	1564m+1564n+1452mn+112	1212m+1212n+1260mn−48
$M_{k}RReZ_{3}$	1440m+1440n+1560mn−120	2552m+2552n+2232mn+320	1880m+1880n+1920mn−40
$M_{k}RBM$	440m+440n+456mn−16	616m+616n+576mn+40	464m+464n+480mn−16
$M_{k}RTM$	776m+776n+816mn−40	1184m+1184n+1104mn+80	944m+944n+984mn−40
$M_{k}RBMH$	1234m+1234n+1320mn−86	2058m+2058n+1842mn+216	1546m+1546n+1590mn−44
$M_{k}RTMH$	2218m+2218n+2412mn−194	3966m+3966n+3534mn+432	3154m+3154n+3246mn−92

**TABLE 5:** Modified reverse degree descriptors for NHC-TM MOFs with reversing parameters k =1, 2, 3.

NHC-TM based MOFs			
TI	k=1	k=2	k=3
$M_{k}RM_{1}$	220m+220n+224mn+216	300m+300n+292mn+308	280m+280n+288mn+272
$M_{k}RM_{2}$	268m+268n+280mn+256	486m+486n+454mn+518	432m+432n+440mn+424
$M_{k}RF$	556m+556n+584mn+528	1004m+1004n+956mn+1052	1024m+1024n+1056mn+992
$M_{k}RHZ$	1092m+1092n+1144mn+1040	1976m+1976n+1864mn+2088	1888m+1888n+1936mn+1840
$M_{k}RReZ_{3}$	1384m+1384n+1504mn+1264	3236m+3236n+2916mn+3556	3192m+3192n+3232mn+3152
$M_{k}RBM$	488m+488n+504mn+472	786m+786n+746mn+826	712m+712n+728mn+696
$M_{k}RTM$	824m+824n+864mn+784	1490m+1490n+1410mn+1570	1456m+1456n+1496mn+1416
$M_{k}RBMH$	1238m+1238n+1324mn+1152	2606m+2606n+2390mn+2822	2540m+2540n+2584mn+2496
$M_{k}RTMH$	2130m+2130n+2324mn+1936	4958m+4958n+4526mn+5390	5180m+5180n+5272mn+5088

**FIGURE 3:**
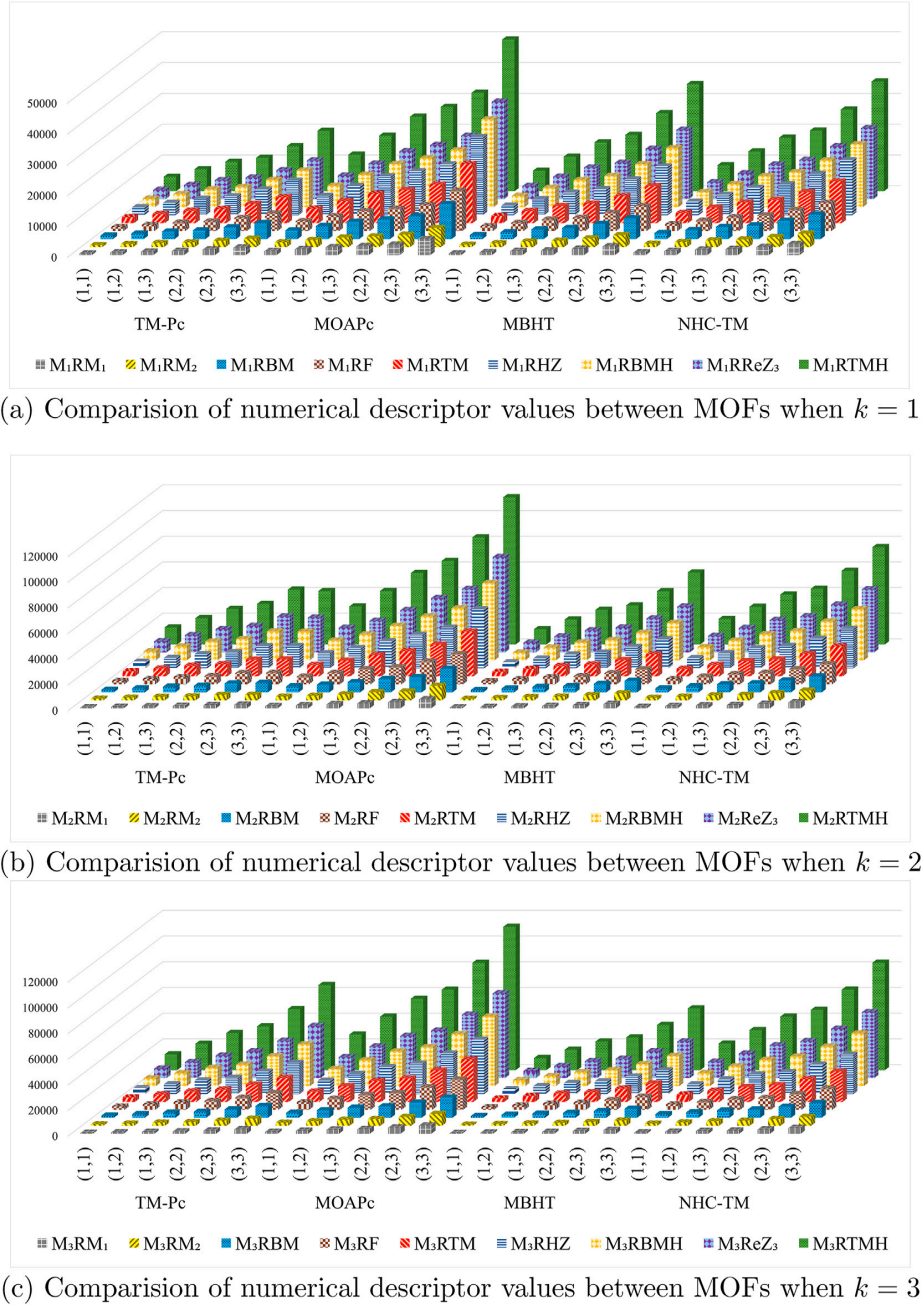
Graphical comparison of numerical descriptors for all MOFs across a range of k values. **(A)** Comparision of numerical descriptor values between MOFs when k=1 . **(B)** Comparision of numerical descriptor values between MOFs when k=2 **(C)** Comparision of numerical descriptor values between MOFs when k=3

We computed the numerical values for the derived topological descriptors of MOFs, which are presented in Tables 6–8 and illustrated in Figure 3. It is clear that the majority of descriptors have largest numerical values for the MOAPc framework, indicating a high level of structural coherence in these MOFs. For all frameworks, the modified reverse degree descriptors $MRM_{1}$ , $MRM_{2}$ , MRBM , MRF , and MRTM are numerically smaller compared to the other descriptors. This suggests that these descriptors should be prioritized when evaluating large frameworks to avoid potential mathematical complexity caused by the exponential growth of other descriptors.

**TABLE 6:** Modified reverse degree based topological descriptors for MOFs when k=1 .

TD	$M_{1}RM_{1}$	$M_{1}RM_{2}$	$M_{1}RF$	$M_{1}RHZ$	$M_{1}RReZ_{3}$	$M_{1}RBM$	$M_{1}RTM$	$M_{1}RBMH$	$M_{1}RTMH$
TM-Pc(1,1)	544	624	1312	2560	3040	1168	1936	2800	4704
TM-Pc(1,2)	852	964	2028	3956	4624	1816	2992	4290	7162
TM-Pc(1,3)	1160	1304	2744	5352	6208	2464	4048	5780	9620
TM-Pc(2,2)	1332	1488	3132	6108	7032	2820	4620	6570	10,902
TM-Pc(2,3)	1812	2012	4236	8260	9440	3824	6248	8850	14,642
TM-Pc(3,3)	2464	2720	5728	11,168	12,672	5184	8448	11,920	19,664
MOAPc(1,1)	1280	1520	3200	6240	7680	2800	4720	6960	11,920
MOAPc(1,2)	1924	2292	4828	9412	11,640	4216	7120	10,526	18,074
MOAPc(1,3)	2568	3064	6456	12,584	15,600	5632	9520	14,092	24,228
MOAPc(2,2)	2892	3456	7284	14,196	17,640	6348	10,740	15,918	27,402
MOAPc(2,3)	3360	4020	8440	16,480	20,640	7380	12,460	18,560	31,980
MOAPc(3,3)	5152	6176	13,024	25,376	31,680	11,328	19,200	28,528	49,232
MBHT (1,1)	564	756	1572	3084	4320	1320	2328	3702	6654
MBHT (1,2)	944	1272	2648	5192	7320	2216	3920	6256	11,284
MBHT (1,3)	1324	1788	3724	7300	10,320	3112	5512	8810	15,914
MBHT (2,2)	1516	2052	4276	8380	11,880	3568	6328	10,130	18,326
MBHT (2,3)	2088	2832	5904	11,568	16,440	4920	8736	14,004	25,368
MBHT (3,3)	2852	3876	8084	15,836	22,560	6728	11,960	19,198	34,822
NHC-TM(1,1)	880	1072	2224	4368	5536	1952	3296	4952	8520
NHC-TM(1,2)	1324	1620	3364	6604	8424	2944	4984	7514	12,974
NHC-TM(1,3)	1768	2168	4504	8840	11,312	3936	6672	10,076	17,428
NHC-TM(2,2)	1992	2448	5088	9984	12,816	4440	7536	11,400	19,752
NHC-TM(2,3)	2660	3276	6812	13,364	17,208	5936	10,088	15,286	26,530
NHC-TM(3,3)	3552	4384	9120	17,888	23,104	7936	13,504	20,496	35,632

**TABLE 7:** Modified reverse degree based topological descriptors for MOFs when k=2 .

TD	$M_{2}RM_{1}$	$M_{2}RM_{2}$	$M_{2}RF$	$M_{2}RHZ$	$M_{2}RReZ_{3}$	$M_{2}RBM$	$M_{2}RTM$	$M_{2}RBMH$	$M_{2}RTMH$
TM-Pc(1,1)	784	1288	2640	5216	8720	2072	3928	6968	13,288
TM-Pc(1,2)	1232	2006	4112	8124	13,444	3238	6118	10,784	20,492
TM-Pc(1,3)	1680	2724	5584	11,032	18,168	4404	8308	14,600	27,696
TM-Pc(2,2)	1932	3120	6396	12,636	20,712	5052	9516	16,674	31,578
TM-Pc(2,3)	2632	4234	8680	17,148	27,980	6866	12,914	22,564	42,664
TM-Pc(3,3)	2976	4288	8864	17,440	27,136	7264	13,152	22,288	41,584
MOAPc(1,1)	1776	2880	5968	11,728	19,232	4656	8848	15,480	29,528
MOAPc(1,2)	2432	3464	8792	15,720	24,664	5896	12,256	20,192	41,624
MOAPc(1,3)	3248	4624	11,744	20,992	32,912	7872	16,368	26,952	55,560
MOAPc(2,2)	3972	6384	13,284	26,052	42,312	10,356	19,668	34,182	65,142
MOAPc(2,3)	4888	6952	17,680	31,584	49,448	11,840	24,632	40,516	83,524
MOAPc(3,3)	7040	11,264	23,488	46,016	74,368	18,304	34,752	60,192	114,656
MBHT (1,1)	708	1140	2412	4692	7656	1848	3552	6174	11,898
MBHT (1,2)	1172	1868	3972	7708	12,440	3040	5840	10,074	19,398
MBHT (1,3)	1636	2596	5532	10,724	17,224	4232	8128	13,974	26,898
MBHT (2,2)	1864	2944	6288	12,176	19,456	4808	9232	15,816	30,432
MBHT (2,3)	2556	4020	8604	16,644	26,472	6576	12,624	21,558	41,466
MBHT (3,3)	3476	5444	11,676	22,564	35,720	8920	17,120	29,142	56,034
NHC-TM(1,1)	1200	1944	4016	7904	12,944	3144	5960	10,424	19,832
NHC-TM(1,2)	1792	2884	5976	11,744	19,096	4676	8860	15,420	29,316
NHC-TM(1,3)	2384	3824	7936	15,584	25,248	6208	11,760	20,416	38,800
NHC-TM(2,2)	2676	4278	8892	17,448	28,164	6954	13,170	22,806	43,326
NHC-TM(2,3)	3560	5672	11,808	23,152	37,232	9232	17,480	30,192	57,336
NHC-TM(3,3)	4736	7520	15,680	30,720	49,216	12,256	23,200	39,968	75,872

**TABLE 8:** Modified reverse degree based topological descriptors for MOFs when k=3 .

TD	$M_{3}RM_{1}$	$M_{3}RM_{2}$	$M_{3}RF$	$M_{3}RHZ$	$M_{3}RReZ_{3}$	$M_{3}RBM$	$M_{3}RTM$	$M_{3}RBMH$	$M_{3}RTMH$
TM-Pc(1,1)	704	1040	2528	4608	7456	1744	3568	6032	12,320
TM-Pc(1,2)	1148	1732	4172	7636	12,536	2880	5904	10,086	20,598
TM-Pc(1,3)	1592	2424	5816	10,664	17,616	4016	8240	14,140	28,876
TM-Pc(2,2)	1860	2856	6828	12,540	20,832	4716	9684	16,686	34,074
TM-Pc(2,3)	2572	3980	9484	17,444	29,128	6552	13,464	23,286	47,550
TM-Pc(3,3)	3552	5536	13,152	24,224	40,640	9088	18,688	32,432	66,224
MOAPc(1,1)	1616	2304	5840	10,448	16,416	3920	8144	13,432	27,688
MOAPc(1,2)	2432	3464	8792	15,720	24,664	5896	12,256	20,192	41,624
MOAPc(1,3)	3248	4624	11,744	20,992	32,912	7872	16,368	26,952	55,560
MOAPc(2,2)	3660	5208	13,236	23,652	37,056	8868	18,444	30,354	62,574
MOAPc(2,3)	4888	6952	17,680	31,584	49,448	11,840	24,632	40,516	83,524
MOAPc(3,3)	6528	9280	23,616	42,176	65,984	15,808	32,896	54,080	111,488
MBHT (1,1)	588	804	2028	3636	5640	1392	2832	4638	9462
MBHT (1,2)	988	1348	3412	6108	9440	2336	4760	7774	15,862
MBHT (1,3)	1388	1892	4796	8580	13,240	3280	6688	10,910	22,262
MBHT (2,2)	1592	2168	5504	9840	15,160	3760	7672	12,500	25,508
MBHT (2,3)	2196	2988	7596	13,572	20,880	5184	10,584	17,226	35,154
MBHT (3,3)	3004	4084	10,396	18,564	28,520	7088	14,480	23,542	48,046
NHC-TM(1,1)	1120	1728	4096	7552	12,768	2848	5824	10,160	20,720
NHC-TM(1,2)	1688	2600	6176	11,376	19,192	4288	8776	15,284	31,172
NHC-TM(1,3)	2256	3472	8256	15,200	25,616	5728	11,728	20,408	41,624
NHC-TM(2,2)	2544	3912	9312	17,136	28,848	6456	13,224	22,992	46,896
NHC-TM(2,3)	3400	5224	12,448	22,896	38,504	8624	17,672	30,700	62,620
NHC-TM(3,3)	4544	6976	16,640	30,592	51,392	11,520	23,616	40,992	83,616

## 4 Comparative study of entropy levels

Consider a subset X that belongs to the union of V(G) and E(G) . Let f be a function that provides structural information, defined on X and mapping to positive real numbers $\left(f:X\to R^{+}\right)$ . Assuming $X=E\left(G\right)=\left\{E_{1}\cup E_{2}\cup \cdots \cup E_{n}\right\}$ , the bond graph entropy of G concerning the topological descriptors function $f\in \left\{M_{k}RTD\right\}$ is given by

$$\begin{array}{ll} I_{f}\left(G\right) & =-\underset{uv\in E\left(G\right)}{\sum }\frac{f\left(u,v\right)}{\sum_{uv\in E\left(G\right)}f\left(u,v\right)}\log\left[\frac{f\left(u,v\right)}{\sum_{uv\in E\left(G\right)}f\left(u,v\right)}\right]\\ & =\log\left[\overset{n}{\underset{i=1}{\sum }}f\left(E_{i}\right)\right]-\frac{1}{\sum_{i=1}^{n}f\left(E_{i}\right)}\log\left[\overset{n}{\underset{i=1}{\prod }}\;{f{\left(u,v\right)}^{f\left(u,v\right)}}^{\left|E_{i}\right|}\right] \end{array}$$

A recent study (Paul et al., 2023) scrutinized the aforementioned expression, involving the replacement of the term $\overset{n}{\underset{i=1}{\prod }}{f{\left(u,v\right)}^{f\left(u,v\right)}}^{\left|E_{i}\right|}$ with $\overset{n}{\underset{i=1}{\prod }}|E_{i}|\cdot \;f{\left(u,v\right)}^{f\left(u,v\right)}$ . Consequently, our study adopts the resulting entropy formula.

$$I_{f}\left(G\right)=\log\left[\overset{n}{\underset{i=1}{\sum }}f\left(E_{i}\right)\right]-\frac{1}{\sum_{i=1}^{n}f\left(E_{i}\right)}\log\left[\overset{n}{\underset{i=1}{\prod }}|E_{i}|\cdot \;f{\left(u,v\right)}^{f\left(u,v\right)}\right]$$

We now illustrate the calculation of the first Zagreb entropy value for the TM-Pc structure. Let G represent the TM-Pc metal organic framework. Upon substitution into the entropy equation, we obtain

$$I_{M_{1}RM_{1}}\left(G\right)=\log\left(M_{1}RM_{1}\right)-\frac{1}{\left(M_{1}RM_{1}\right)}\;\log\left[\overset{n}{\underset{i=1}{\prod }}|E_{i}|\cdot {\left(M_{k}R\left(d_{u}\right)+M_{k}R\left(d_{v}\right)\right)}^{\left(M_{k}R\left(d_{u}\right)+M_{k}R\left(d_{v}\right)\right)}\right]$$

By substituting the edge partition classes specified in Table 1, we can express entropy for TM-Pc (m,n) based on $M_{1}$ descriptor as follows.

$$\begin{array}{cc} I_{M_{1}RM_{1}}\left(\text{TM}-\text{Pc}\left(m,n\right)\right) & =\log\left(172mn+136\left(m+n\right)+100\right)\\ & \;-\frac{1}{172mn+136\left(m+n\right)+100}\log\left[\left(4\left(m+n+2\right)\right)\times {\left(3+3\right)}^{\left(3+3\right)}\right.\\ & \;\times \left(16mn+12\left(m+n\right)+8\right)\times {\left(3+2\right)}^{\left(3+2\right)}\times \left(20mn+10\left(m+n\right)\right)\\ & \;\left.\times {\left(2+2\right)}^{\left(2+2\right)}\times \left(4\left(mn+m+n+1\right)\right)\times {\left(2+1\right)}^{\left(2+1\right)}\right] \end{array}$$

Assuming m=n=3 , we acquire

$$\begin{array}{c} I_{M_{1}RM_{1}}\left(\text{TM}-\text{Pc}\left(3,3\right)\right)=\log\left(2464\right)\\ -\frac{1}{2464}\log\left[1007769600000\times 110100480\right]\\ I_{M_{1}RM_{1}}\left(\text{TM}-\text{Pc}\left(3,3\right)\right)=3.39164070349\\ -\left(0.00040584415\times 20.0451504658\right)\\ I_{M_{1}RM_{1}}\left(\text{TM}-\text{Pc}\left(3,3\right)\right)=3.38350 \end{array}$$

The above-outlined approach is implemented to calculate the entropy levels for MOFs based on the reverse degree indices. We would like to point out that recent literature includes a comprehensive comparative analysis across diverse chemical structures such as graphene, graphyne, graphdiyne, $C_{4}$ C_8_ nanosheets, honeycomb network, γ -graphyne, kekulene structures, zigzag graphyne nanoribbons, and carbon nanosheets (Govardhan et al., 2024; Rahul et al., 2022; Raja and Anuradha, 2024; Peter and Clement, 2024; Kavitha et al., 2021) based on degree indices. Therefore, the current investigation focuses on modified reverse degree-based entropy levels to provide robust measures, thus assessing their effectiveness and offering insights for potential structure developments. A comparative analysis is presented in Tables 7–10, highlighting the impact of varying the reverse parameter k on entropy levels across different MOFs.

In evaluating the entropy levels from Tables 9–12, we explore dynamic variations across MOFs at k=1,2,3 . Notably, entropy consistently increases at k=3 compared to k=1 and 2, with $M_{3}RTMH$ showing highest levels. However, for MOAPc and MBHT, the measures show minimal difference at k=2 and k=3 . To assess the complexity, the direct comparison is challenging due to edge variability. Thus, we incorporate scaled bond-wise entropy measures (BIs) , normalizing entropy by considering the number of edges in the frameworks (Arockiaraj et al., 2023d). The bond-wise entropy BIs offers a detailed perspective on structural characteristics and stability dynamics within MOFs. For example, TM-Pc(1,3) has $I_{M_{3}RTMH}=4.4325$ , and |E(TM-Pc(1,3))| =260, then the bond-wise entropy is measured for TM-Pc(1,3) by the following formula.

$$BI_{M_{3}RTMH}\left(\text{TM}-\text{Pc}\left(1,3\right)\right)=\frac{I_{M_{3}RTMH}\left(\text{TM}-\text{Pc}\left(1,3\right)\right)}{\left|E\left(\text{TM}-\text{Pc}\left(1,3\right)\right)\right|}=\frac{4.4325}{260}=0.017048$$

Now, we consider the bond ranges with reasonably acceptable classes and compute the bond-wise entropy for the tri-Zagreb harmonic index, which are represented in Table 11.

**FIGURE 4:**
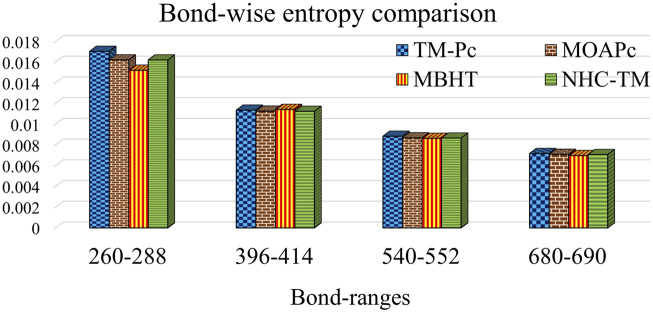
Graphical comparison of bond-wise entropy levels among MOFs with different bond ranges.

Table 13 and Figure 4 provide a comparison of bond-wise entropies across different structures, maintaining consistent |E(G)| ranges among all MOFs. Bond-wise entropy, representing entropy per bond, provides intuitive comparisons for each molecular structure. Across all frameworks, TM-Pc exhibits the highest normalized entropy at all dimensions, while NHC-TM and MBHT display lower values, suggesting lesser complexity. Additionally, as the frameworks expand in dimensions (m,n) , the normalized entropies of TM-Pc, MOAPc, NHC-TM and MBHT converge, indicating comparable complexity levels across maximum-dimensional MOFs, regardless of bonding patterns.

**TABLE 9:** Comparing entropy levels for TM-Pc (m,m) based MOFs between k=1 , k=2 and k=3 .

TD	TM-Pc (m,m) based MOF											
	k=1				k=2				k=3			
	m=1	m=2	m=3	m=4	m=1	m=2	m=3	m=4	m=1	m=2	m=3	m=4
$I_{M_{k}RM_{1}}$	2.703	3.110	3.384	3.590	2.860	3.271	3.465	3.754	2.815	3.256	3.543	3.757
$I_{M_{k}RM_{2}}$	2.760	3.157	3.426	3.630	3.070	3.477	3.622	3.956	2.978	3.441	3.735	3.953
$I_{M_{k}RF}$	3.077	3.478	3.748	3.953	3.374	3.786	3.936	4.267	3.359	3.818	4.110	4.327
$I_{M_{k}RHZ}$	3.360	3.765	4.037	4.242	3.661	4.078	4.228	4.561	3.611	4.079	4.374	4.592
$I_{M_{k}RReZ_{3}}$	3.428	3.823	4.089	4.292	3.875	4.288	4.416	4.768	3.810	4.296	4.597	4.818
$I_{M_{k}RBM}$	3.028	3.434	3.705	3.911	3.272	3.685	3.851	4.167	3.200	3.658	3.950	4.167
$I_{M_{k}RTM}$	3.242	3.645	3.916	4.121	3.541	3.957	4.106	4.439	3.504	3.968	4.262	4.480
$I_{M_{k}RBMH}$	3.395	3.795	4.064	4.267	3.781	4.196	4.333	4.677	3.723	4.201	4.500	4.720
$I_{M_{k}RTMH}$	3.612	4.011	4.279	4.482	4.052	4.469	4.601	4.951	4.025	4.509	4.809	5.029

**TABLE 10:** Comparing entropy measures for MOAPc (m,m) based MOFs between k=1 , k=2 and k=3 .

TD	MOAPc (m,m) based MOF											
	k=1				k=2				k=3			
	m=1	m=2	m=3	m=4	m=1	m=2	m=3	m=4	m=1	m=2	m=3	m=4
$I_{M_{k}RM_{1}}$	3.087	3.452	3.706	3.903	3.230	3.590	3.842	4.037	3.192	3.556	3.810	4.006
$I_{M_{k}RM_{2}}$	3.158	3.527	3.784	3.982	3.438	3.795	4.046	4.240	3.343	3.708	3.963	4.159
$I_{M_{k}RF}$	3.477	3.850	4.108	4.305	3.750	4.112	4.364	4.559	3.745	4.112	4.368	4.565
$I_{M_{k}RHZ}$	3.762	4.137	4.396	4.594	4.039	4.402	4.655	4.850	3.994	4.363	4.619	4.816
$I_{M_{k}RReZ_{3}}$	3.842	4.228	4.490	4.691	4.250	4.611	4.863	5.056	4.186	4.556	4.812	5.009
$I_{M_{k}RBM}$	3.422	3.791	4.048	4.245	3.644	4.004	4.256	4.451	3.573	3.938	4.194	4.390
$I_{M_{k}RTM}$	3.643	4.017	4.275	4.474	3.919	4.281	4.534	4.729	3.887	4.255	4.511	4.708
$I_{M_{k}RBMH}$	3.804	4.185	4.446	4.645	4.157	4.519	4.771	4.965	4.101	4.470	4.726	4.923
$I_{M_{k}RTMH}$	4.029	4.417	4.681	4.882	4.432	4.797	5.050	5.244	4.412	4.783	5.040	5.237

**TABLE 11:** Comparing entropy levels for NHC-TM (m,m) based MOFs between k=1 , k=2 and k=3 .

TD	NHC-TM (m,m) based MOF											
	k=1				k=2				k=3			
	m=1	m=2	m=3	m=4	m=1	m=2	m=3	m=4	m=1	m=2	m=3	m=4
$I_{M_{k}RM_{1}}$	2.919	3.287	3.543	3.740	3.055	3.416	3.669	3.864	3.031	3.397	3.653	3.849
$I_{M_{k}RM_{2}}$	2.998	3.374	3.633	3.832	3.261	3.618	3.869	4.062	3.221	3.585	3.839	4.035
$I_{M_{k}RF}$	3.309	3.689	3.950	4.150	3.569	3.933	4.186	4.381	3.591	3.960	4.216	4.413
$I_{M_{k}RHZ}$	3.595	3.979	4.241	4.441	3.858	4.224	4.477	4.672	3.854	4.223	4.480	4.677
$I_{M_{k}RReZ_{3}}$	3.685	4.082	4.350	4.552	4.065	4.428	4.680	4.873	4.081	4.449	4.705	4.901
$I_{M_{k}RBM}$	3.256	3.632	3.891	4.089	3.467	3.828	4.080	4.275	3.435	3.801	4.056	4.253
$I_{M_{k}RTM}$	3.475	3.858	4.120	4.320	3.738	4.102	4.356	4.551	3.743	4.111	4.368	4.565
$I_{M_{k}RBMH}$	3.642	4.034	4.299	4.500	3.974	4.338	4.590	4.784	3.983	4.351	4.607	4.803
$I_{M_{k}RTMH}$	3.866	4.268	4.536	4.740	4.245	4.613	4.866	5.061	4.289	4.659	4.915	5.112

**TABLE 12:** Comparing entropy levels for MBHT (m,m) based MOFs between k=1 , k=2 and k=3 .

TD	MBHT (m,m) based MOF											
	k=1				k=2				k=3			
	m=1	m=2	m=3	m=4	m=1	m=2	m=3	m=4	m=1	m=2	m=3	m=4
$I_{M_{k}RM_{1}}$	2.712	3.165	3.447	3.655	2.810	3.255	3.532	3.738	2.737	3.189	3.470	3.679
$I_{M_{k}RM_{2}}$	2.833	3.295	3.579	3.788	3.010	3.450	3.726	3.930	2.870	3.322	3.603	3.811
$I_{M_{k}RF}$	3.142	3.611	3.897	4.107	3.325	3.776	4.055	4.261	3.265	3.725	4.008	4.218
$I_{M_{k}RHZ}$	3.425	3.899	4.187	4.398	3.604	4.059	4.339	4.546	3.512	3.975	4.259	4.468
$I_{M_{k}RReZ_{3}}$	3.560	4.047	4.339	4.552	3.804	4.258	4.536	4.741	3.695	4.160	4.444	4.653
$I_{M_{k}RBM}$	3.070	3.533	3.818	4.027	3.214	3.662	3.939	4.145	3.104	3.560	3.842	4.051
$I_{M_{k}RTM}$	3.307	3.779	4.066	4.276	3.488	3.941	4.220	4.426	3.406	3.868	4.152	4.361
$I_{M_{k}RBMH}$	3.498	3.980	4.270	4.482	3.716	4.170	4.449	4.654	3.614	4.077	4.361	4.571
$I_{M_{k}RTMH}$	3.741	4.233	4.526	4.739	3.989	4.449	4.730	4.936	3.915	4.384	4.670	4.879

**TABLE 13:** Bond-wise entropy comparison among MOFs with similar bond ranges.

$BI_{M_{3}RTMH}$							
Bond ranges based on edge variability of metal organic frameworks							
G(m,n)	260-288	G(m,n)	396-414	G(m,n)	540-552	G(m,n)	680-690
TM-Pc(1,3)	0.017048	TM-Pc(2,3)	0.011366	TM-Pc(1,7)	0.00885	TM-Pc(1,9)	0.007184
MOAPc(1,1)	0.016219	MOAPc(1,2)	0.011272	MOAPc(1,3)	0.008694	MOAPc(1,4)	0.007103
MBHT(2,2)	0.015223	MBHT(2,3)	0.011438	MBHT(3,3)	0.008648	MBHT(3,4)	0.006982
NHC-TM(1,2)	0.016215	NHC-TM(2,2)	0.011253	NHC-TM(2,3)	0.008673	NHC-TM(2,4)	0.007082

## 5 Estimating graph energy with regression models

Graph energy significantly influences electronic properties, impacting chemical reactions, materials science, energy conservation, and optoelectronics. Moreover, graph energy directly relates to total π -electronic energy. In this study, we use integrated software newGRAPH for computing the adjacency matrix from molecular structures (Stevanović et al., 2021). Let A(G) represent the adjacency matrix of metal organic framework G with order m . The eigenvalues of A(G) , denoted $\lambda_{1},\lambda_{2},\ldots ,\lambda_{m}$ , constitute graphs spectrum (Gutman, 1978). The energy $E_{\pi }\left(G\right)$ of the graph G is determined by the absolute sum of the eigenvalues of the spectrum.

$$E_{\pi }\left(G\right)=\overset{m}{\underset{i=1}{\sum }}|\lambda_{i}|$$

In the literature, studies focused on distance, degree, degree-sum based topological descriptors, and energies for structures such as zeolite frameworks, benzenoid hydrocarbons, polyhex nanotubes, hypercubes, and porous graphene (Taherpour and Mohammadinasab, 2010; Hayat et al., 2020; Hayat et al., 2021a; Balasubramanian, 2023; Govardhan and Roy, 2023; Hayat et al., 2021b). However, in this study, we investigate the relationship between graph energy $E_{\pi }\left(G\right)$ and various modified reverse degree descriptors. High correlation coefficient values between these descriptors and $E_{\pi }\left(G\right)$ underscore the expanded importance of graph energy beyond its conventional use in Hückel molecular orbital theory (Gutman et al., 2017).

Calculating graph energy for higher-order dimensions (m,n) of metal organic frameworks using newGRAPH software can be complex as it involves generating the adjacency matrix for higher dimensions. However, we develop statistical models to accurately predict energy for these higher orders by integrating all four distinct frameworks into a unified dataset. Energy values for fixed graph frameworks of (m,n) are computed using newGRAPH software detailed in Table 12. We derive linear and quadratic regression equations for predicting graph energy based on topological descriptor values computed from the respective frameworks, as shown in Tables 6–8. Additionally, a comparison between the linear and quadratic regression equations is utilized to assess their predictive capacity against the regression models.

### 5.1 Regression models for prediction of spectral properties

In this section, we conduct a comprehensive correlation analysis of the topological descriptors and spectral characteristics of various metal organic frameworks.

The predictive regression models are proposed using the following equations.

Linear equation:Y=aX+b

where Y is the spectral property to be predicted, X is the respective topological descriptor, a represents the slope coefficient and b represents as constant coefficient of the regression line such that the standard error (SE) should be low, and the F-value should be high. Correlation coefficient (r) measures the robustness of linear relationships and the goodness of fit. The values of the correlation coefficients, along with statistical metrics like $r^{2}$ and adjusted $r^{2}$ , are examined and discussed.

We perform a regression analysis to identify the best predicting topological descriptor for the considered metal organic frameworks. This analysis explores the relationship between the graph energy values provided in Table 14 and the molecular descriptors listed in Tables 6–8, emphasizing descriptors that display strong positive correlations. To improve predictive accuracy, we analyze individual MOFs separately. As an example, we illustrate the regression analysis for the NHC-TM (m,n) framework to first choose the best descriptor for predicting graph energy values provided in Table 14, using the NHC-TM (m,n) framework descriptor values presented in Table 6 for the reversing parameter k=1 as outlined in Table 15.

Table 15, shows that the values of r , $r^{2}$ , and adjusted $r^{2}$ are similar for all topological descriptors. When considering other parameters for effective prediction modeling, again the Zagreb index $\left(M_{1}RM_{1}\right)$ stands out due to its higher F value and lower standard error (S.E.) , suggesting it as the optimal predictive model. We further compare the predictive ability for NHC-TM(1,4) Zagreb descriptor-based linear regression models between the reversing parameters k=1,2 and three is presented in Table 16.

Table 16 demonstrates that the regression equation for k=3 predicts energy values more accurately compared to other k values. Similarly, for all other mentioned metal organic frameworks (MOFs), the Zagreb descriptor $\left(M_{3}RM_{1}\right)$ serves as the best predictor for the graph energies. The detailed results for the mentioned frameworks are presented in Table 17.

Utilizing the regression models from Table 17, we predict the graph energies of higher-dimensional metal organic frameworks and present a comparison between the actual graph energies and the predicted energies using the optimal model and present in the Table 18.

**FIGURE 5:**
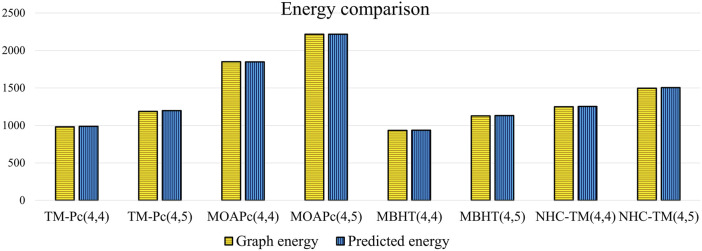
Comparison of predicted energy of MOFs.

Table 18 and Figure 5 illustrate that the predictive model provides energy values closely matching the actual graph energies computed by the newGRAPH software. This capability enables accurate prediction of graph energy values for higher-dimensional distinct MOFs.

**TABLE 14:** Energy values for MOFs obtained from newGRAPH.

MOF (G)	$E_{\pi }$	MOF (G)	$E_{\pi }$	MOF (G)	$E_{\pi }$	MOF (G)	$E_{\pi }$
TM-Pc(1,1)	133.489	MOAPc(1,1)	298.584	MBHT(1,1)	118.364	NHC-TM(1,1)	202.508
TM-Pc(1,2)	210.027	MOAPc(1,2)	446.769	MBHT(1,2)	196.369	NHC-TM(1,2)	302.791
TM-Pc(1,3)	286.562	MOAPc(1,3)	594.955	MBHT(1,3)	274.374	NHC-TM(1,3)	403.073
TM-Pc(2,2)	329.709	MOAPc(2,2)	668.495	MBHT(2,2)	312.924	NHC-TM(2,2)	452.234
TM-Pc(2,3)	449.389	MOAPc(2,3)	890.221	MBHT(2,3)	429.479	NHC-TM(2,3)	602.009
TM-Pc(3,3)	612.212	MOAPc(3,3)	1185.487	MBHT(3,3)	584.584	NHC-TM(3,3)	800.796

**TABLE 15:** Regression equations for MOFs correlating energy and the topological descriptors when k=1 .

TD	Equations	r	$r^{2}$	Adj $\left(r^{2}\right)$	S.E	F -values
$M_{1}RM_{1}$	0.224 $\left(M_{1}RM_{1}\right)$ +6.399	1.000	1.000	1.000	0.721	443554.09
$M_{1}RM_{2}$	0.181 $\left(M_{1}RM_{2}\right)$ +10.264	1.000	1.000	1.000	1.071	201083.98
$M_{1}RF$	0.087 $\left(M_{1}RF\right)$ +11.058	1.000	1.000	1.000	1.143	176547.28
$M_{1}RHZ$	0.044 $\left(M_{1}RHZ\right)$ +10.669	1.000	1.000	1.000	1.108	187971.83
$M_{1}RReZ_{3}$	0.034 $\left(M_{1}RReZ_{3}\right)$ +16.093	1.000	1.000	1.000	1.599	90158.66
$M_{1}RBM$	0.100 $\left(M_{1}RBM\right)$ +8.538	1.000	1.000	1.000	0.915	275689.91
$M_{1}RTM$	0.059 $\left(M_{1}RTM\right)$ +10.800	1.000	1.000	1.000	1.120	183992.04
$M_{1}RBMH$	0.038 $\left(M_{1}RBMH\right)$ +13.734	1.000	1.000	1.000	1.385	120139.99
$M_{1}RTMH$	0.022 $\left(M_{1}RTMH\right)$ +16.683	1.000	1.000	1.000	1.653	84413.05

**TABLE 16:** Comparing graph energy for NHC-TM(1,4) with $M_{k}RM_{1}$ predicted values for k=1,2 , and 3.

k values	Regression Equations	$M_{k}RM_{1}$	Predicted values	Graph energy $\left(E_{\pi }\right)$
k=1	0.224 $\left(M_{1}RM_{1}\right)$ +6.399	2212	501.887	503.356
k=2	0.169 $\left(M_{2}RM_{1}\right)$ -4.33	2976	498.614	503.356
k=3	0.175 $\left(M_{3}RM_{1}\right)$ +7.925	2824	502.125	503.356

**TABLE 17:** Optimal regression models for metal organic frameworks.

MOFs	Optimal Models	r	$r^{2}$	adj $\left(r^{2}\right)$	S.E	F
TM-Pc (m,n)	0.168 $\left(M_{3}RM_{1}\right)$ +17.263	1.000	1.000	1.000	1.572	60106.05
MOAPc (m,n)	0.180 $\left(M_{3}RM_{1}\right)$ +7.747	1.000	1.000	1.000	0.704	1021886.24
MBHT (m,n)	0.193 $\left(M_{3}RM_{1}\right)$ +5.781	1.000	1.000	1.000	0.676	306276.288
NHC-TM (m,n)	0.175 $\left(M_{3}RM_{1}\right)$ +7.925	1.000	1.000	1.000	0.859	312470.73

**TABLE 18:** Comparing graph energy of MOFs using optimal models.

MOFs	$E_{\pi }$	Predicted $\left(E_{\pi }\right)$	MOFs	$E_{\pi }$	Predicted $\left(E_{\pi }\right)$
TM-Pc(4,4)	981.0009	988.303	TM-Pc(4,5)	1186.967	1197.967
MOAPc(4,4)	1849.559	1847.347	MOAPc(4,5)	2216.154	2216.707
MBHT(4,4)	933.343	936.813	MBHT(4,5)	1126.997	1132.129
NHC-TM(4,4)	1248.390	1253.925	NHC-TM(4,5)	1496.661	1504.525

## 6 Conclusion

We have conducted a comprehensive analysis by calculating modified reverse degree topological descriptors for four types of MOFs. Simultaneously, we have performed a detailed assessment of entropy levels for each MOF and compared these levels with the bond-wise scaled entropy approach across all frameworks. Moreover, we have presented an optimal linear regression model for predicting the graph energy of distinct structural frameworks, aiming to reduce the computational complexity of software and produce results in polynomial time. The graph theoretical and statistical methods explored in this study can enhance machine learning applications in computational chemistry and QSAR/QSPR studies for material advancements.

## Data Availability

The original contributions presented in the study are included in the article/supplementary material, further inquiries can be directed to the corresponding author.
